# MXtalTools: A Toolkit for Machine Learning on Molecular Crystals

**DOI:** 10.1021/acs.jcim.5c02868

**Published:** 2026-03-24

**Authors:** Michael Kilgour, Mark E. Tuckerman, Jutta Rogal

**Affiliations:** † Department of Chemistry, 5620 New York University, New York, New York 10003, United States; ‡ Department of Physics, 5894 New York University, New York, New York 10003, United States; § 525571 NYU−ECNU Center for Computational Chemistry at NYU Shanghai, Shanghai 200062, China; ∥ Simons Center for Computational Physical Chemistry at New York University, New York, New York 10003, United States; ⊥ Initiative for Computational Catalysis, Flatiron Institute, New York, New York 10010, United States

## Abstract

We present MXtalTools, a flexible Python package for the data-driven modeling of molecular crystals, facilitating machine learning studies of the molecular solid state. MXtalTools comprises several classes of utilities: (1) synthesis, collation, and curation of molecule and crystal data sets, (2) integrated workflows for model training and inference, (3) crystal parametrization and representation, (4) crystal structure sampling and optimization, (5) end-to-end differentiable crystal sampling, construction, and analysis. Our modular functions can be integrated into existing workflows or combined and used to build novel modeling pipelines. MXtalTools leverages CUDA acceleration to enable high-throughput crystal modeling. The Python code is available open-source on our GitHub page, with detailed documentation on ReadTheDocs.

## Graphical abstract

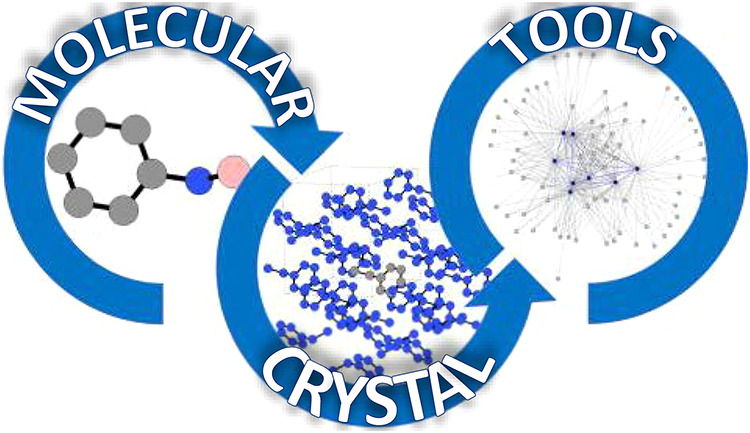

## 1 Introduction

Molecular crystals are a diverse class of materials with many applications across numerous industries, including pharmaceuticals, agrochemicals, electronics, and energetic materials, and significant efforts have been devoted to understanding and engineering their properties. While there is a large and mature software universe focused on data-driven modeling atomistic/inorganic materials, molecular crystals are distinct in important ways which call for purpose-built solutions. For example, an accurate treatment of crystal symmetry operations applied to molecules is essential.

One of the primary computational challenges in the field of molecular crystals is crystal structure prediction (CSP), the determination of the most likely crystal structures for a given molecule, generally including structure generation, optimization, and ranking.^3^ Related tasks, such as crystal representation, classification, and property prediction, are also of interest to many researchers.

Efficient machine learning on molecular crystals requires a set of minimal capabilities, including GPU acceleration, efficient batched operations, and support for automated gradient flow (autograd) through crystal building and analysis operations. Existing packages for computationally guided molecular crystal structure prediction,^4–8^ while very useful, all lack one or more of these critical features, and hence are not suited to high throughput molecular crystals modeling. MXtalTools (MXT) is an open-source Python code written specifically to address this shortfall, taking advantage of these capabilities to enable a wide range of modeling tasks, including crystal scoring, density prediction, optimization, and more.

A subtle yet important capability provided by native PyTorch^9^ integration is that many of MXtalTools’ workflows are end-to-end differentiable and fast enough to be included on-the-fly in training loops. This enables immense flexibility in the types of workflows users can deploy, including crystal generation, optimization, or scoring, on any differentiably computable crystal or molecule property.

## 2 Components

The primary modules of MXT are summarized in Figure 1. MXT is mainly structured around the construction and manipulation of molecule and crystal data objects, via MolData and MolCrystalData classes. These data objects are created, parametrized, filtered, and collated through our database methods, for subsequent use in modeling workflows.

**Figure 1:**
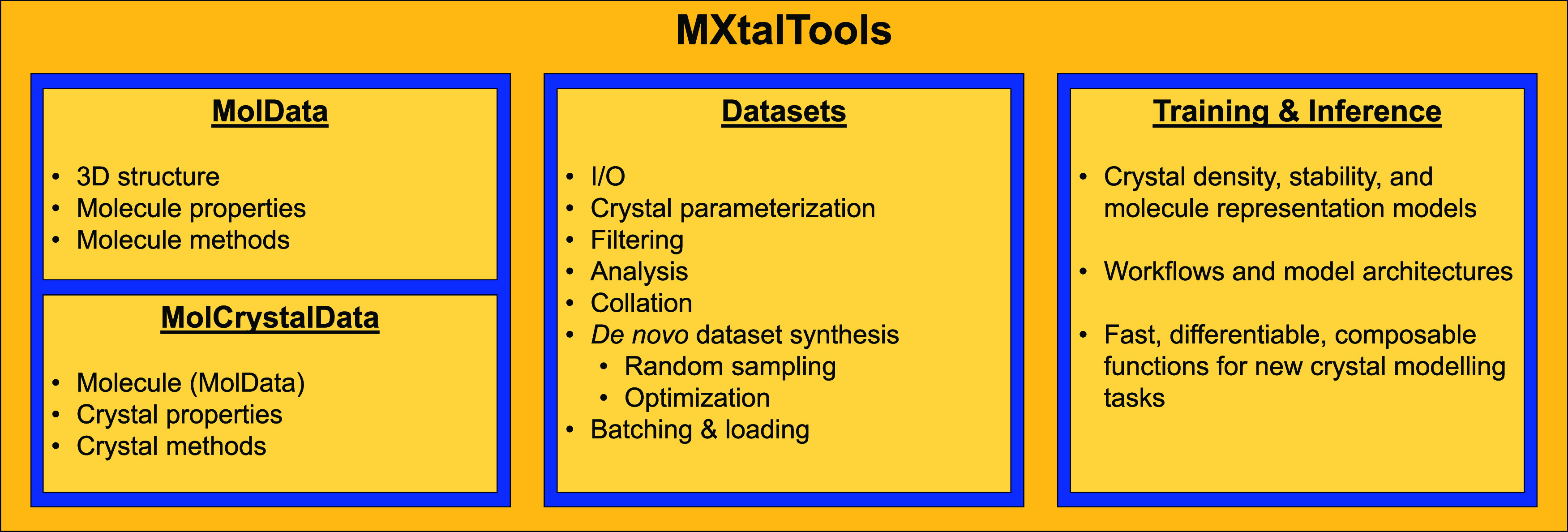
Major components of MXtalTools.

### 2.1 Data Classes

Inheriting batching and indexing behavior from the PyTorch Geometric Data and Batch classes, molecule and crystal objects can be collated and manipulated batch-wise for efficiency. Their instantiation and basic usage is illustrated with the following examples.

Molecules can be initialized either by explicit enumeration of the atoms, or built from SMILES strings^10^ (via RDKit^11^).
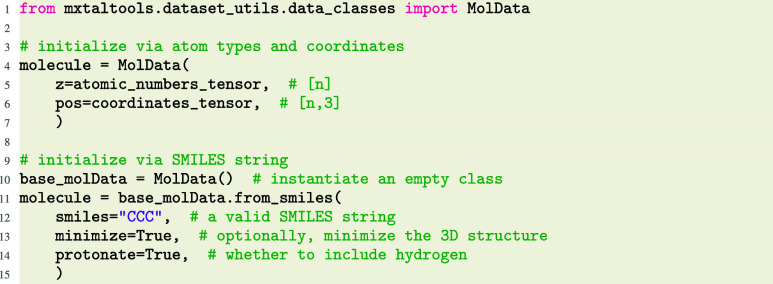
Upon initialization, molecule geometric properties such as mass, volume, and radius are automatically computed. Main attributes and methods are enumerated in Figure 2.

**Figure 2:**
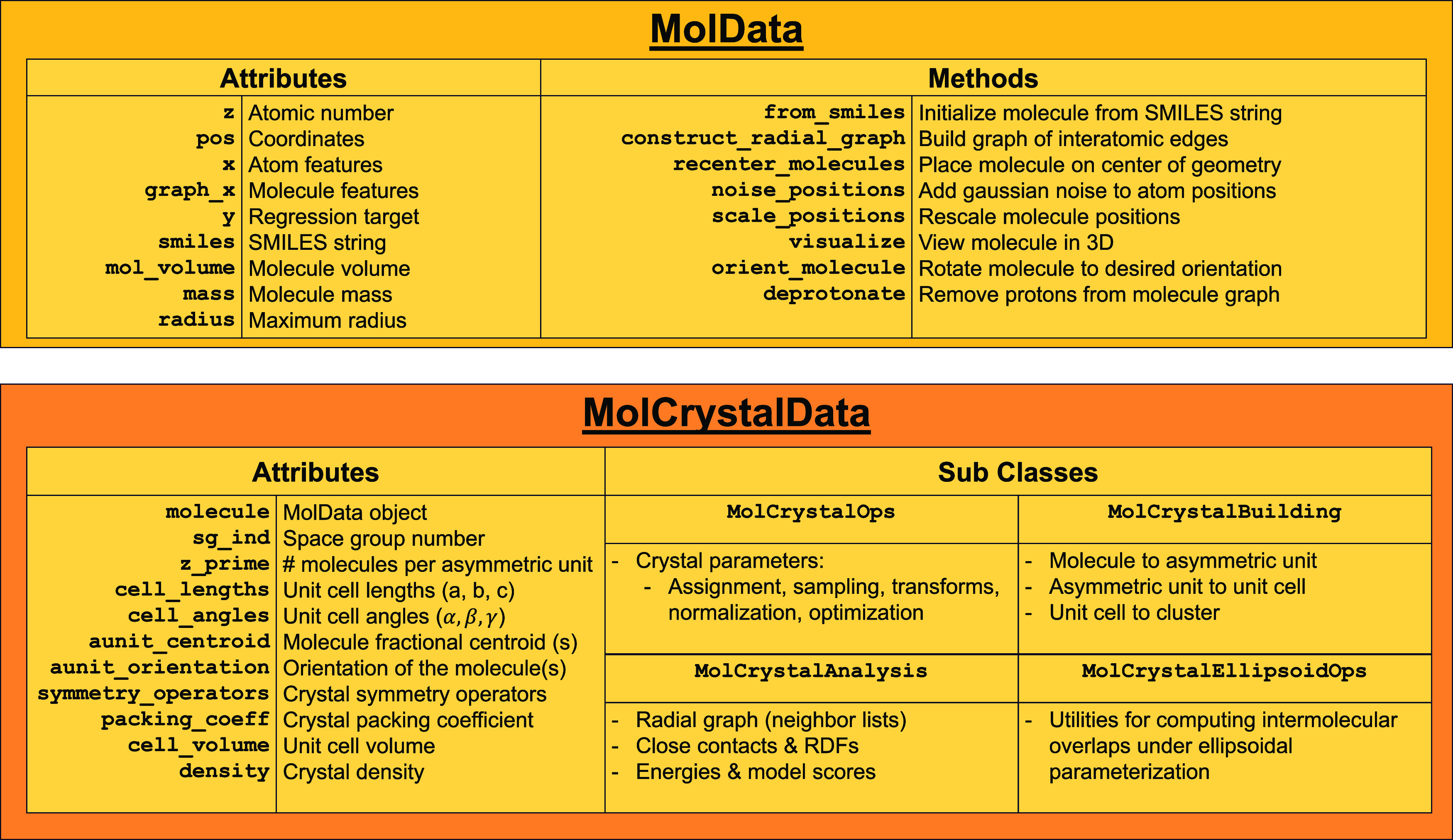
Class summary diagram for MolData and MolCrystalData objects.

Molecular crystals are built from the combination of molecules, as instantiated above, and crystal parameters, defined in detail in the Supporting Information (SI). MolCrystalData inherits class methods and properties from the original MolData, and adds all the utilities required for parametrizing, constructing, and analyzing molecular crystals.
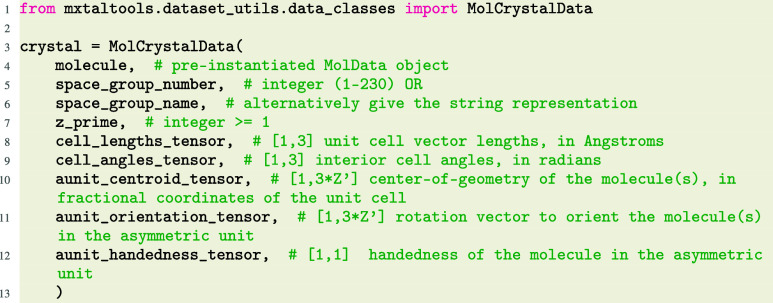


Standard symmetry settings/operations are assumed, but can be overwritten when necessary. Crystals are represented in a reduced-dimensional format via parametrization of molecules as rigid objects that are posed and oriented with respect to a “standard” initial state, defined as having the center of geometry at the origin and the molecule principal inertial axes aligned with the Cartesian axes. Combined with information on the box and symmetry information, these reduced degrees of freedom comprise the “crystal parameters”. Important attributes and methods are shown in the class diagram in Figure 2.

Crystal parameters can be hard-coded, randomly generated, or extracted from unit cells of existing crystal samples (e.g., those extracted from databases). We provide examples in Section 3.

### 2.2 Data Set Construction

A typical MXtalTools training data set is constructed by (1) reading source files, typically crystallographic information files (cif’s) for crystals and XYZ (Cartesian coordinate) files or SMILES for molecules, (2) computing the necessary sample features, (e.g., mass, volume, crystal parameters), and (3) instantiating each sample in a MolData or MolCrystalData object. For crystal data sets, extensive filtering and processing is required to ensure data quality, typically rejecting >25% of samples, depending on the data desired. In common crystal data sets, such as the Cambridge Structural Database (CSD),^12^ large numbers of crystal entries are either irrelevant for our purposes, such as disordered or polymeric structures, or have a serious flaw, such as missing atoms, or containing obviously unphysical structures. Our crystal parametrization scheme requires a clean definition of the molecular conformer (which may be rigid or flexible), the unit cell, and the crystal symmetry operations. Therefore, crystals from trusted data sources can be instantiated directly from prebuilt unit cells and the appropriate symmetry operators. The workflows and filtering conditions are detailed in the SI.

### 2.3 Model Training

We currently provide workflows to train models for molecule properties, crystal properties, molecule autoencoding, and molecule and crystal property prediction from fixed embeddings (see workflow diagrams in the SI). We have trained models for the crystal density/unit cell volume/crystal packing coefficient, given only the molecule graph, as well as the 20 QM9/QM9s^13,14^ quantum mechanical properties of small organic molecules. Additional property prediction models are easily trainable, provided sufficiently large synthetic or experimental training data sets. These could include material properties such as the Young’s modulus, brittleness, plasticity, elasticity, and compressibility, for which data sets could plausibly be generated with accurate general potentials^15,16^ and appropriate simulation protocols.^17^ We plan also to train a model for high precision molecular volume prediction in the near future.

Given the modular nature of our training and reporting utilities, adding new workflows is straightforward. Training runs are controlled via yaml configuration files, and logged to the Weights & Biases online platform through our Logger class, which also collects and processes convergence and evaluation statistics.

### 2.4 Models

Included under the models directory is a library of custom machine learning models, modules, and utilities. Through a combination of standard scalar/invariant neural network operations and O(3) equivariant operations, as detailed in Ref 18, we construct:

1. Standard multilayer perceptrons (MLPs, or feedforward fully connected neural networks), with skip connections, normalization, and dropout options.
2. Equivariant MLP models (EMLPs), for modeling higher-order tensor properties such as dipole or quadrupole moments.
3. Standard and equivariant graph neural networks (GNNs), correspondingly modeling scalars and vectors, respectively. The GNN models can be used on single molecules or clusters carved out of molecular crystal supercells. The clusters represent the crystalline environments required in the computation of the embedding for the canonical conformer in the molecular crystal graph.^19^

A typical crystal scalar property prediction model might comprise 4 graph convolution layers and 4 MLP layers, all with 512 feature channels, layer normalization, and dropout probability of 0.25.

All our graph models are built on a BaseGraphModel class, which assumes an input in the form of a point cloud with atom types and positions, and optionally molecule-wide properties, and can be mixed and matched with MLP models as appropriate for a given modeling task.

To demonstrate the efficiency of our primary workflows, as well as the advantages of GPU acceleration, we include in the SI a table of mean walltimes for several representative MXT operations, for multiple batch sizes, on CPU and GPU hardware.

MXtalTools is designed and optimized for maximum efficiency with large batches and CUDA GPU acceleration. Parallel CPU execution is viable for ‘offline’ or preprocessing tasks, but not currently recommended in model training.

## 3 Examples

In this section, we will demonstrate the deployment of some of our utilities and pretrained models on practical analyses. As of the time of writing, the crystal volume and score models are trained on the *Z*′ = 1 CSD structures, excluding polymers, porous structures, nonstandard symmetries, and erroneous structures, as outlined in.^19^ The Mo3ENet molecule autoencoder^18^ was trained on randomly generated conformers of “QM9-like” molecules from the QM9 and ZINC22 data sets,^13,20,21^ meaning they contain H, C, N, O and F, with up to 9 heavy atoms. All demonstrated workflows use standard envelope functions or sufficiently large interaction cutoffs, ensuring stable and differentiable optimization.

### 3.1 Molecule Representation

As a step toward modeling molecular crystals via a low-dimensional embedding rather than with an all-atom approach, we developed the Mo3ENet (molecular O(3) equivariant encoder net) autoencoder model.^18^ Mo3ENet converts molecule point clouds into representations that are equivariant to rotation and inversion, and that provably contain complete information on atom positions and types.

Generating a molecule embedding is done by passing a batch of molecules to the autoencoder model, and retrieving the equivariant “vector” embedding, and its rotationally invariant subrepresentation, the “scalar” embedding.
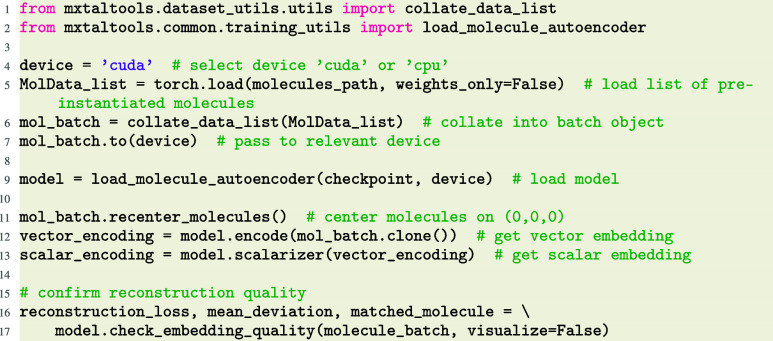


We can confirm the embedding is of sufficiently high quality by computing its reconstruction loss and mean deviation from the target structure, and even visualize the molecule and reconstruction together. If the reconstruction is accurate, the embedding necessarily contains complete information about the molecular point cloud.

### 3.2 Crystal Density Prediction

Following our work in,^19^ we illustrate a workflow for predicting the unit cell volume for a given molecule, without any direct knowledge of the crystal structure. The quantity to be regressed can be cast in several equivalent ways: crystal packing coefficient, $C_{P}=\frac{V_{\mathrm{mol}}\cdot Z}{V_{\mathrm{cell}}}$ where *Z* is the symmetry multiplicity of the space group (number of molecules per unit cell), crystal density ρ, or asymmetric or unit cell volume, *V*_aunit_, *V*_cell_. The crystal packing coefficient is generally preferred as it is unitless and largely independent of molecule size and space group. There is no universally accepted definition of molecule volume, and as such no general method to compute it. To get consistent volume estimates from given *C*_*p*_ model, one must therefore use the molecule volume calculator used when preparing the training data.

Running inference with a pretrained model is straightforward and the corresponding workflow would be very similar for any molecule property prediction task. One has only to load and batch the molecules, load the model, and then evaluate the desired property:
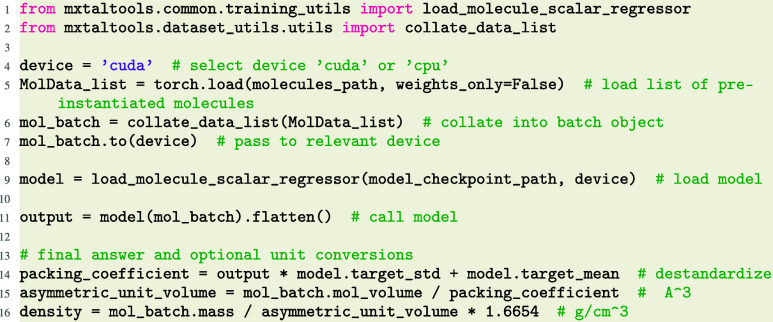


### 3.3 Crystal Building and Analysis

A core component of our codebase is the construction and analysis of molecular crystals. Most straightforwardly, one may load prebuilt crystals, build explicit convolution clusters, and analyze them:
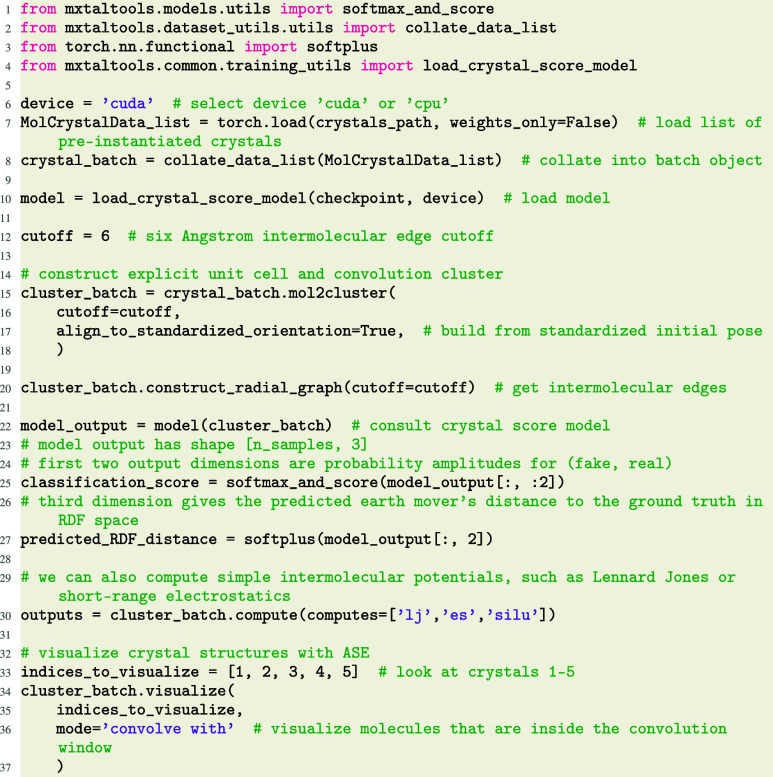


The MolCrystalData interface also allows for easy crystal analysis, including Lennard–Jones energies, a short-range electrostatic potential, and a repulsion-softened Lennard-Jones-style energy (“SiLU” potential, see definition in SI). We also provide interfaces for the popular MACE and UMA machine learned interatomic potentials (MLIPs),^15,16,22^ and can easily include additional MLIPs with pre-existing Atomic Simulation Environment (ASE)^23^ interfaces. Sample visualization is handled via the use the ASE package’s visualize.view function.

Our crystal score models (MolXtalNet-S) are trained on experimental crystal structures from the CSD and randomly generated crystal “fakes”. MolXtalNet-S predicts two values: (1) a classification score, representing the confidence the crystal **is** the experimentally observed structure, and (2) a distance metric quantifying the distance in radial distribution space between a given crystal and the experimental structure. This radial distribution function earth mover’s distance (RDF EMD) is defined in detail in the Supporting Information (SI).

Our crystal building and analysis functions are all differentiable, enabling the computation of gradients through crystal analysis and construction, back to the crystal parameters or even a generating function for said parameters, by calling output.mean­().backward­() on the outputs. We demonstrate a practical application in Section 4.

## 4 Case Study

To show how one can combine MXtalTools modules into useful workflows, we present a case study of a basic crystal structure prediction via local optimization of randomly seeded crystal structures. The purpose of this case study is not competitive CSP benchmarking, but rather a demonstration of what types of workflows can be constructed by composition of MXT operations. In particular, we employ a Lennard-Jones potential energy function as an inexpensive, though primarily illustrative, approximation of the true potential energy surface. By design, our crystal building, analysis and optimization operations are agnostic to space group and molecule geometry, treating the former as lists of symmetry operators to apply, and the latter as rigid bodies to be translated and rotated in the asymmetric unit. As such, the optimization workflow is robust to such choices, even in large, mixed batches. This workflow will identify likely crystal structures of a given molecule, 1,3,4,5,6,8-hexafluoronaphthalene-2,7-diamine, CSD ID: DAFMUV (inset in Figure 3 panel (a)). DAFMUV has an experimentally known crystal structure^24^ in space group number 33: Pna21, serving as a reference for the predicted structures. Because our workflows operate on rigid molecules and DAFMUV is itself fairly rigid, we use the experimental conformer directly as input.

**Figure 3:**
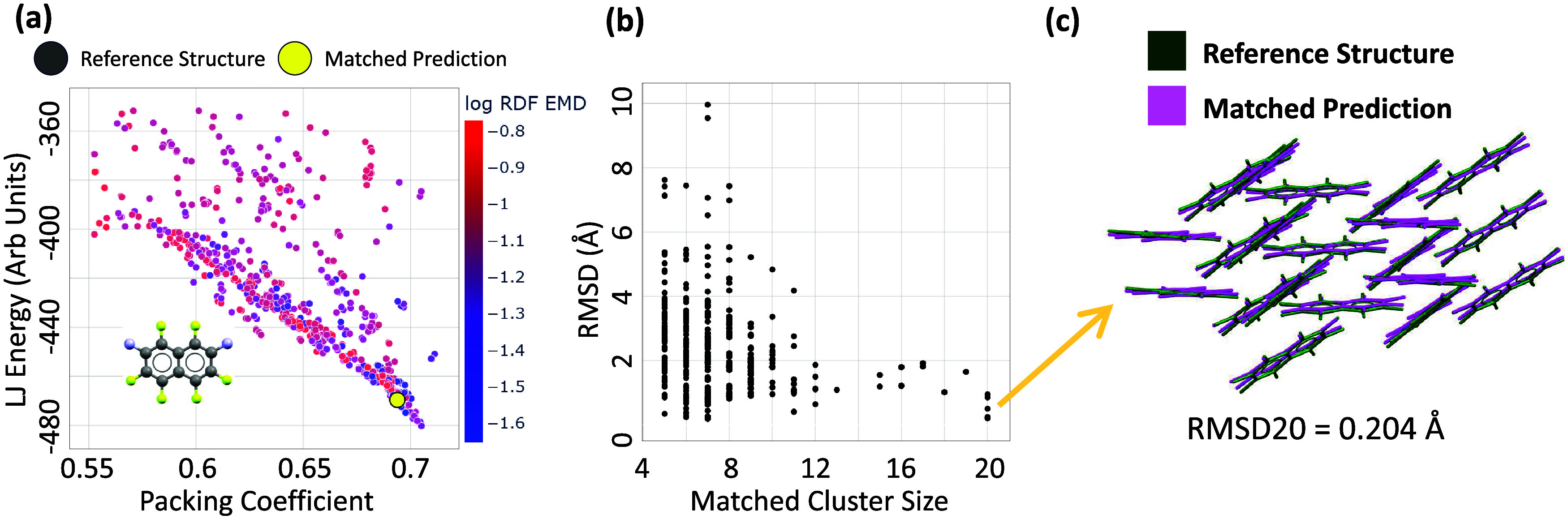
(a) Results of a crystal search run, showing the intermolecular LJ energy as a function of density (crystal packing coefficient), colors indicate the log of the RDF EMD between each sample and the experimental crystal structure, (b) RMSD and number of matched molecules for 755 structures, (c) the closest 20/20 matched cluster with RMSD of 0.204Å.

We leverage the end-to-end differentiability afforded by PyTorch integration, and optimize the crystal parameters directly via backpropagation through molecule posing, crystal building, and scoring/analysis. Our crystal search module can optimize crystals on any differentiable PyTorch function, from simple energies to pretrained crystal score models such as MolXtalNet-S. For this case study, we opted for simple energy functions to showcase the entire workflow with minimal computational cost.

We begin our search (procedure detailed in the SI) by estimating the likely density of the crystal using MolXtalNet-D, following the example workflow in Section 3.2, and receive an estimated crystal packing coefficient of **0.699**, within 1% agreement with the reference value of **0.694**.

Samples are initialized for optimization with random crystal parameters, and densities set at the target value. Random sampling of molecular crystal parameters for realistic densities typically yields crystals with intermolecular overlap. The too short distances between molecules result in extremely high energy “jammed” structures, which often fail to optimize to reasonable local minima.

We therefore optimize in two stages, first based on a soft-repulsion potential (details in the SI), the squared deviation of the density from the predicted value, and an auxiliary loss to prevent cell vectors from getting too long. The softened interatomic repulsion allows molecules with severe overlap to slide past one another, while still equilibrating to a minimally overlapping local minimum. In the second stage, a short local refinement with respect to the LJ energy and the target density is performed. For simplicity, we omit further refinements, but users can replace the LJ energy or do subsequent optimizations on desired energy functions, including MolXtalNet-D, or pretrained MLIPs.

Panel (a) in Figure 3 shows the energy vs density plot for a batch of 940 optimized crystal structures, with 60 very poor quality structures excluded from an original 1000. The search found a number of structures near the expected experimental density with comparable and even lower energies than the experimental structure. To determine if these lower energy structures are truly more stable than the experimental one, structural refinement and energy evaluations with more accurate energy functions would be needed. Structures similar in densities and energy to the experimental target structure have usually, though not always, the smallest EMD in RDF space, indicating their packing patterns are in good agreement with the experimental one.

In panel (b) of Figure 3, we show the root-mean-square deviation (RMSD) of the generated structures to the experimental one is shown for 755 out of 1000 structures, focusing on samples with packing coefficients between 0.6 and 0.8. RMSD values and number of matched molecules were computed using the COMPACK algorithm^25^ (details in the SI). The COMPACK analysis confirms that our search yielded many structures with good local structural agreement with the experimental polymorph. In panel (c), we visualize the closest match, with an RMSD20 of 0.204Å. The Lennard-Jones potential seems to prefer slightly higher-densities than the experimental structure, yet within 1000 generated samples we were able to find five 20/20 cluster matches.

This case study and the examples in Section 3 demonstrate complicated multistep workflows that can be easily performed with just a few MXtalTools functions. Improvements or modularization can be incorporated seamlessly, for example, a generative model could be substituted for random sampling for initial candidate structures, genetic optimization, Monte Carlo, or other optimization schemes could be combined with gradient descent, and any learned model or autograd-friendly property could be used for scoring and ranking. This flexibility and amenability to both novel experimentation and efficient implementation are the core strengths of this codebase.

## 5 Summary and Outlook

### 5.1 Use Cases

We have introduced our software package, MXtalTools, containing several utilities for the construction, analysis, and modeling of molecular crystals. Particularly time-saving are our crystal data set curation pipeline, our crystal building and analysis methods, and our automated training and inference workflows. The modules form the core utilities for MXT users when developing their own tools and methods. MXT’s general end-to-end differentiability also offers promising opportunities for novel crystal sampling and optimization approaches.

Our tools provide significant support for researchers pursuing a variety of objectives, for example:

1. Experimentalists or computational chemists can easily install the MXtalTools package and estimate the density of a given molecular crystal, based on their molecule(s) of study.
2. Molecular ML scientists can integrate our Mo3ENet molecular embedding models into their workflows, using the equivariant representation as a stand-in for molecules themselves.
3. Computational molecular crystals researchers can use our analysis tools and crystal scoring models to analyze and optimize their candidate structures.
4. Molecular crystal ML researchers can mix and match our modular toolkit to construct custom workflows for novel modeling tasks, including backpropagation through all steps of crystal construction and modeling.

### 5.2 Limitations and Future Directions

MXtalTools currently supports homomolecular (identical molecules and conformers) crystals, for integer *Z*′, and general Wyckoff positions. Molecules are generally assumed to be rigid, though torsional flexibility may be added in a future update. To explore different conformers, one must reinitialize MolData objects with those conformations directly. Unique asymmetric unit parametrization is currently only possible in space groups where the asymmetric unit is an easily defined parallelepiped. This includes most space groups below 99 and a heterogeneous set above, covering >99% of all organic and organometallic molecular crystals in the CSD. The relevant groups are explicitly defined in constants/asymmetric_units.py, and listed in the SI.

Extensions to all space groups, explicitly flexible molecules, and cocrystals, are mostly technically straightforward and planned for the future implementations. Integration of further new models, including generative models for crystals, will come as such new capabilities are developed.

## Supplementary Material



## Data Availability

With the exception of Cambridge Crystallographic Data Centre (CCDC)^26^ software, all the software components required to run MXtalTools are available free of charge, under BSD-3 license. Detailed installation instructions are available on our GitHub README page, with detailed documentation on ReadTheDocs. Crystal cif file cleaning and filtering, as well as COMPACK packing analysis both require the CSD Python API, requiring an active paid license to run. Crystals can be loaded without the CSD Python API, if they come from a trusted source, and users are willing to skip filtering and cleaning. The Cambridge Structural Database^12^ likewise requires a paid license to access in bulk. MXtalTools is tested on Linux and Windows with Python ≥ 3.10.

## Notes

Efficiency benchmarks, computational and mathematical details, workflow diagrams, algorithmic details, and an example script for our case study in Section 4 (PDF)
