# Supplementary material for: MXtalTools: A Toolkit for Machine Learning on Molecular Crystals
Source: J Chem Inf Model. 2026 Mar 24;66(7):3402–8. doi: 10.1021/acs.jcim.5c02868 (PMC13080994; doi:10.1021/acs.jcim.5c02868)
Supplement: Supplementary file 1 [file ci5c02868_si_001.pdf]

# Supporting Information: MXtalTools: A Toolkit for Machine Learning on Molecular Crystals

**Michael Kilgour**[1]* **Mark E. Tuckerman**[1,2,3,4], **Jutta Rogal**[5]

[1]Department of Chemistry, New York University, New York, NY 10003, USA
[2]Department of Physics, New York University, New York, NY 10003, USA
[3]NYU–ECNU Center for Computational Chemistry at NYU Shanghai, Shanghai 200062, China
[4]Simons Center for Computational Physical Chemistry at New York University, New York, NY 10003, USA
[5]Initiative for Computational Catalysis, Flatiron Institute, New York, NY 10010, USA

## S1 Efficiency Benchmark

| Device | Batch size | Unit cell build (ms) | Cluster build (ms) | Neighbor list + LJ (ms) | 100 Local Opt Steps (s) |
|---|---|---|---|---|---|
| CPU | 10 | 5 | 13 | 12 | 7 |
| CPU | 100 | 8 | 64 | 86 | 27 |
| CPU | 1000 | 27 | 468 | 721 | 194 |
| CUDA | 10 | 7 | 6 | 6 | 6 |
| CUDA | 100 | 7 | 13 | 6 | 11 |
| CUDA | 1000 | 11 | 73 | 20 | 62 |

Figure S1: Results of benchmarking experiment, showing mean walltimes for each operation on a batch of random $Z' = 1$ molecular crystals from the CSD. Unit cell build, cluster build, and neighbor-list + LJ statistics were averaged over 10 repeats.

In Figure S1 we present the results of a runtime benchmark for the core MXtalTools crystal building and scoring workflows, and an end-to-end local optimization on the LJ energy. The experiments were done on a laptop with an Intel Core Ultra 9 275HX CPU and NVIDIA GeForce RTX 5080 GPU. Our crystal construction and analysis tools are purpose-built to be run in very large parallel operations on GPU, and we see from the benchmark that the most expensive steps, explicit cluster instantiation and neighbor list construction, are dramatically more efficient on CUDA than CPU, especially for larger batches.

## S2 RDF Distance Calculation

For the estimation of crystal similarity, we compute for each sample the radial distribution (RDF) from 0-6 Å, for all unique pairs of atom types (elements) in the crystal. These pairwise RDFs describe the local neighborhood about a given molecule.

To garner a usable metric from these local 'fingerprints', we compute the earth mover's distance between pairs of crystals' RDFs, and sum the element pair contributions according to their probability mass. This corresponds qualitatively to the distance atoms would need to move in order to transform from one crystal to another.

*Corresponding author: michael.kilgour@nyu.edu

Preprint. Under review.

Stepwise, we compute the RDF distance between crystal 1 and 2 for element pair $(i, j)$ (for example, carbon and nitrogen), via the 1D earth movers distance along the radial direction, with $N$ discrete bins $r$, between normalized radial density functions. Crystal radial density histograms, $R_m^{i,j}(r)$, with bin width $\Delta r$, are normalized according to

$$\bar{R}_m^{i,j}(r) = \frac{R_m^{i,j}(r)}{\sum_r R_m^{i,j}(r) + \epsilon}, \tag{S1}$$

for epsilon a small stability factor. 1D pairwise earth mover's distances are then computed as

$$EMD_{1,2}^{i,j} = \Delta r \sum_{k=0}^{N} \left| \sum_{r=0}^{k} \bar{R}_1^{i,j}(r) - \sum_{r=0}^{k} \bar{R}_2^{i,j}(r) \right|, \tag{S2}$$

with $N$ the total number of radial bins. The crystal-crystal RDF EMD is then aggregated by averaging over atom pairs

$$EMD_{1,2} = \frac{1}{\mathcal{A}} \sum_{i,j \in \mathcal{A}} EMD_{1,2}^{i,j}, \tag{S3}$$

for $\mathcal{A}$ the set of atom pairs with non-vanishing density, corresponding to the elements present in either crystal.

## S3 Workflows

We show in Figures S2 and S3 outlines of the workflows for dataset construction and modelling, respectively, including the rules for filtering from crystal datasets.

Dataset filtering is necessary to avoid corruption of training data with obviously unphysical structures, of merely undesired ones. Examples of the former include data entries with missing or overlapping atoms, or missing or extra atoms or molecules. There are also cases where the given crystal symmetry operations do not align with the stated space group or $Z$ values. As an extra guard, when processing a new dataset we attempt to instantiate a conformer for every molecule using RDKit. If RDKit does not recognize the molecule as valid, the crystal is filtered out. Examples of the latter may include cocrystals, organometallics, porous structures, or polymers, depending on the purpose of the study. Overall filtration rates vary database-to-database, and also between crystal types and entry ages. Rates above 25% are common in general. If users are confident that a new dataset contains realistic molecules and valid symmetry operations, filtering can be skipped.

## S4 SiLU Potential

Due to the steep $1/r^{12}$ increase of the standard Lennard-Jones repulsive term at short distances, it can sometimes be an unstable optimization target, especially in situations with large unphysical interatomic overlaps. We define a simple and robust interatomic potential that roughly maintains the shape and position of the LJ potential well, with linear repulsion for nonzero van der Waals overlaps. While unphysically soft, this potential never explodes / is always well-behaved, even for poor crystal structures. Unlike the Buckingham potential, this energy also requires no fine-tuning of constants, and does not diverge near zero.

The SiLU potential, so named from the use of the 'Sigmoid Linear Unit' activation function, common in neural network modelling, between two atoms is given as

$$E_{SiLU}(r) = \frac{7}{25} SiLU\left(-4R\left(r - \sigma R\right)\right), \tag{S4}$$

for SiLU the sigmoid linear unit, $\frac{x}{1+e^{-x}}$, r the interatomic distance, $\sigma$ the sum of atoms' van der Waals Radii, and $R$ a scaling factor which shifts the repulsive onset to the left (softening) or right (hardening), set to 1 by default.

A visual comparison to the standard Lennard-Jones potential is given in Figure S4. We see that the location and rough shape of the minima agree, though the SiLU attraction decays faster. This is actually advantageous in the typical role of this potential, that is, coarse structure optimization, where the shorter range of the potential allows for shorter interatomic cutoffs, 6Å vs. 10 for full LJ.

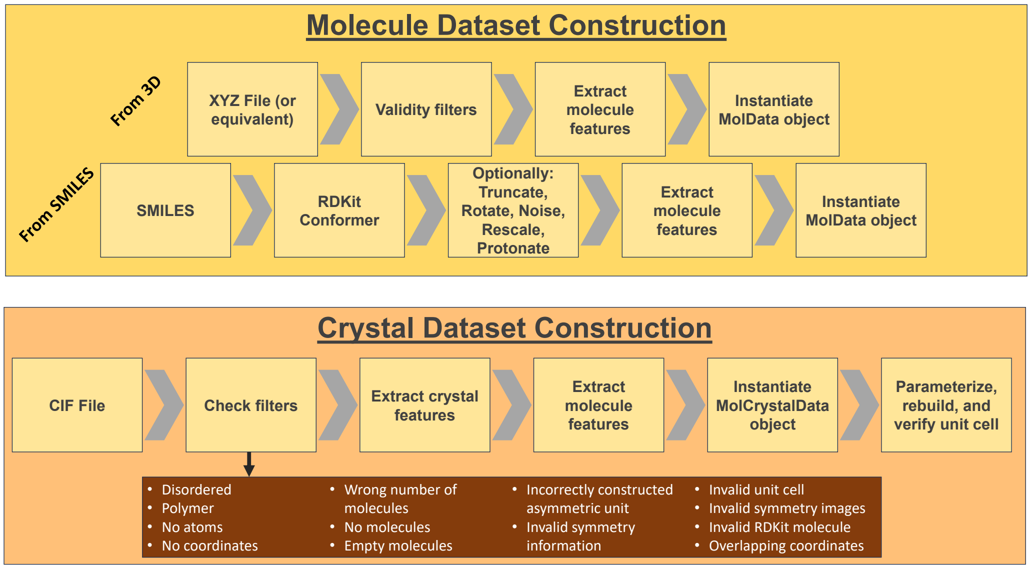


Figure S2: Workflows for molecule and crystal data point creation.

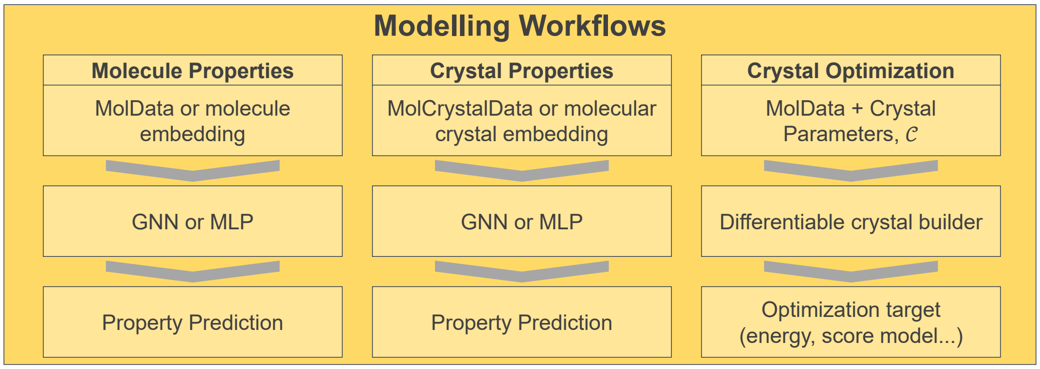


Figure S3: MXtalTools main modelling workflows.

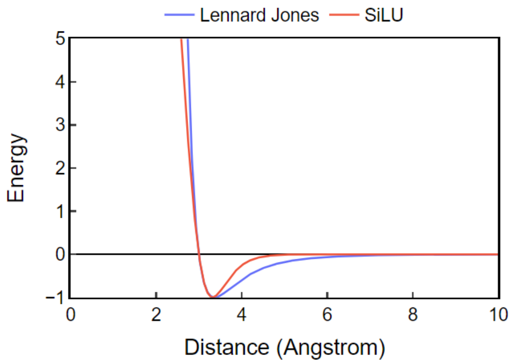


Figure S4: Visual comparison of SiLU and Lennard-Jones potentials near the potential minimum, with $\sigma$ taken as 3 Å, $R = 1$.

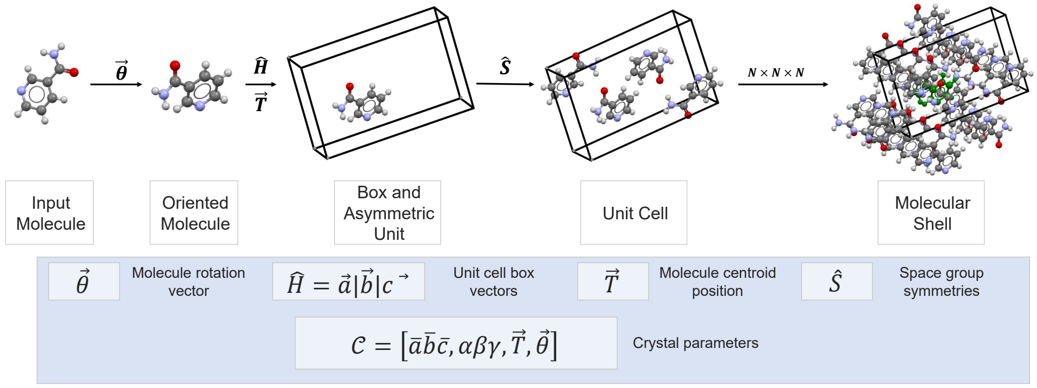


Figure S5: Graphic representation of our crystal building pipeline for a $Z' = 1$ molecular crystal, combining the molecule, position and orientation in the asymmetric unit (pose), crystal symmetry operations (space group) and box vectors to generate the unit cell. For subsequent analysis, we pattern an $N \times N \times N$ supercell and carve out a cluster surrounding the asymmetric unit.

## S5 Crystal Parameterization

A rigid $Z' = 1$ molecular crystal placed on a general Wyckhoff position is completely described by the molecular conformation, space group symmetry, and 12 crystal parameters, $\mathcal{C}$. These parameters correspond to cell vector lengths ($\bar{a}$,$\bar{b}$,$\bar{c}$), internal angles ($\alpha$,$\beta$,$\gamma$), the position of the molecule centroid in unit cell fractional coordinates $\vec{T} = (u, v, w)$, and the molecule orientation defined against a standardized orientation by a rotation vector $\vec{\theta} = (x, y, z)$. The box vectors are defined in cartesian coordinates as

$$\vec{a} = (\bar{a}, 0, 0) \tag{S5}$$

$$\vec{b} = (\bar{b}\cos\gamma, \bar{b}\sin\gamma, 0) \tag{S6}$$

$$\vec{c} = \left(\bar{c}\cos\beta, \bar{c}\frac{\cos\alpha - \cos\beta\cos\gamma}{\sin\gamma}, \bar{c}\sqrt{1 - \cos^2\beta - \left(\frac{\cos\alpha - \cos\beta\cos\gamma}{\sin\gamma}\right)^2}\right), \tag{S7}$$

with $V$ the unit cell volume. The cell parameters and crystal building process is outlined in Figure S5. Box parameters are generally given explicitly in crystal .cif files, and pose parameters can be extracted using MXtalTools data processing utilities.

## S6 COMPACK Parameters

The following parameters were used for the COMPACK structure comparison in the case study in the main text.

distance tolerance = 0.4

angle tolerance = 40

allow molecular differences = True

packing shell size = 20

## S7 Supported space groups

MXtalTools currently supports crystal building analysis either for custom, user-supplied symmetry operations, or standard symmetry operations for molecules on general Wyckhoff positions, with well-defined asymmetric units. We consider 'well-defined' asymmetric units to be those whose canonical asymmetric units are simple parallelepipeds. Currently, the supported space groups are:

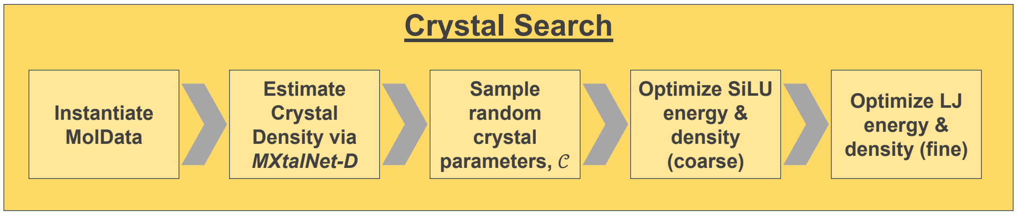


Figure S6: Workflow diagram for our crystal search case study.

| | | | | | |
|---|---|---|---|---|---|
| 1:P1 | 22:F222 | 43:Fdd2 | 64:Cmce | 85:P4/n | 114:P-421c |
| 2:P-1 | 23:I222 | 44:Imm2 | 65:Cmmm | 86:P42/n | 115:P-4m2 |
| 3:P2 | 24:I212121 | 45:Iba2 | 66:Cccm | 87:I4/m | 116:P-4c2 |
| 4:P21 | 25:Pmm2 | 46:Ima2 | 67:Cmme | 88:I41/a | 117:P-4b2 |
| 5:C2 | 26:Pmc21 | 47:Pmmm | 68:Ccce | 89:P422 | 118:P-4n2 |
| 6:Pm | 27:Pcc2 | 48:Pnnn | 69:Fmmm | 90:P4212 | 119:I-4m2 |
| 7:Pc | 28:Pma2 | 49:Pccm | 70:Fddd | 91:P4122 | 120:I-4c2 |
| 8:Cm | 29:Pca21 | 50:Pban | 71:Immm | 92:P41212 | 122:I-42d |
| 9:Cc | 30:Pnc2 | 51:Pmma | 72:Ibam | 93:P4222 | 124:P4/mcc |
| 10:P2/m | 31:Pmn21 | 52:Pnna | 73:Ibca | 94:P42212 | 126:P4/nnc |
| 11:P21/m | 32:Pba2 | 53:Pmna | 74:Imma | 95:P4322 | 128:P4/mnc |
| 12:C2/m | 33:Pna21 | 54:Pcca | 75:P4 | 96:P43212 | 130:P4/ncc |
| 13:P2/c | 34:Pnn2 | 55:Pbam | 76:P41 | 97:I422 | 131:P42/mmc |
| 14:P21/c | 35:Cmm2 | 56:Pccn | 77:P42 | 98:I4122 | 133:P42/nbc |
| 15:C2/c | 36:Cmc21 | 57:Pbcm | 78:P43 | 103:P4cc | 135:P42/mbc |
| 16:P222 | 37:Ccc2 | 58:Pnnm | 79:I4 | 104:P4nc | 137:P42/nmc |
| 17:P2221 | 38:Amm2 | 59:Pmmn | 80:I41 | 105:P42mc | 141:I41/amd |
| 18:P21212 | 39:Aem2 | 60:Pbcn | 81:P-4 | 106:P42bc | 142:I41/acd |
| 19:P212121 | 40:Ama2 | 61:Pbca | 82:I-4 | 109:I41md | |
| 20:C2221 | 41:Aea2 | 62:Pnma | 83:P4/m | 110:I41cd | |
| 21:C222 | 42:Fmm2 | 63:Cmcm | 84:P42/m | 112:P-42c | |

## S8 Crystal Search

The procedure for the crystal search case study follows the workflow outlined in Figure S6. The Python code required to run such a search is provided below and the .yaml config file used for the optimization is also provided. We find the Rprop (resilient propagation) optimizer to be both the most robust and fastest converging out of the standard PyTorch optimizers, on the space defined by our crystal degrees of freedom. This makes intuitive sense, as Rprop was developed specifically to optimize rugged and anisotropic landscapes.

```

import os
import subprocess
import sys

import numpy as np
import torch
from tqdm import tqdm

from examples.crystal_search_reporting import batch_compack, density_funnel,
    compack_fig
from mxtaltools.analysis.crystal_rdf import compute_rdf_distance

# add MXtalTools to path by relative reference
sys.path.insert(0, os.path.abspath("../"))

import torch.nn.functional as F
from mxtaltools.common.training_utils import load_crystal_score_model
from mxtaltools.dataset_utils.utils import collate_data_list
```

```python
from mxtaltools.models.utils import softmax_and_score

torch.set_grad_enabled(False)

device = 'cuda'
dafmuv_path = "datasets/DAFMUV.pt"
mini_dataset_path = '../mini_datasets/mini_CSD_dataset.pt'
score_checkpoint = r"../checkpoints/crystal_score.pt"
density_checkpoint = r"../checkpoints/cp_regressor.pt"
opt_path = r"opt_outputs/DAFMUV.pt"

"""
Load crystal score model
"""
score_model = load_crystal_score_model(score_checkpoint, device).to(device)
score_model.eval()

"""
Load and analyze reference crystal
"""
dafmuv_data = torch.load(dafmuv_path, weights_only=False)
ref_crystal_batch = collate_data_list(dafmuv_data).to(device)
ref_computes, ref_cluster_batch = ref_crystal_batch.analyze(
    computes=['lj'], return_cluster=True, cutoff=10, supercell_size=10)
model_output_ref = score_model(ref_cluster_batch.to(device), force_edges_rebuild=True).cpu()
ref_score = softmax_and_score(model_output_ref[:, :2]).cpu()
ref_pred_rdfemd = F.softplus(model_output_ref[:, 2]).cpu()
rdf, bin_edges, _ = ref_cluster_batch.compute_rdf()
ref_rdf = rdf.cpu()

ref_lj_energy = ref_computes['lj'].cpu()
ref_cp = ref_cluster_batch.packing_coeff.cpu()

"""
Run optimization via standalone script & config
"""
subprocess.run(["python", "run_search.py", "--input", "dafmuv_example.yaml"], check=True)

"""
Analyze optimized samples
"""
opt_sample_list = torch.load(opt_path, weights_only=False)
batch_size = 25
num_batches = len(opt_sample_list) // batch_size + int((len(opt_sample_list) % batch_size) > 0)
opt_score, opt_pred_rdfemd, opt_rdfs, opt_lj_energy, opt_cp = [], [], [], [], []
for batch_idx in tqdm(range(num_batches)):
    opt_crystal_batch = collate_data_list(opt_sample_list[batch_size * batch_idx:batch_size * (1 + batch_idx)]).to(
        device)
    computes, opt_cluster_batch = opt_crystal_batch.analyze(
        computes=['lj','rdf'], return_cluster=True, cutoff=10, supercell_size=10
    )

    model_output = score_model(opt_cluster_batch.to(device), force_edges_rebuild=True).cpu()
    opt_score.append(softmax_and_score(model_output[:, :2]).cpu())
    opt_pred_rdfemd.append(F.softplus(model_output[:, 2]).cpu())
    opt_rdfs.append(computes['rdf'][0].cpu())
    opt_lj_energy.append(computes['lj'].cpu())
    opt_cp.append(opt_crystal_batch.packing_coeff.cpu(

opt_score = torch.cat(opt_score)
```

```python
opt_pred_rdfemd = torch.cat(opt_pred_rdfemd)
opt_rdfs = torch.cat(opt_rdfs)
opt_lj_energy = torch.cat(opt_lj_energy)
opt_cp = torch.cat(opt_cp)

"""
Compute true RDF distances
"""
rdf_dists = compute_rdf_distance(ref_rdf[0], opt_rdfs, bin_edges.to('cpu'))

"""
COMPACK analysis
"""
best_sample_inds = torch.argwhere((opt_cp > 0.6) * (opt_cp < 0.8)).squeeze()
matches, rmsds = batch_compack(best_sample_inds, opt_sample_list, ref_crystal_batch)

all_matched = np.argwhere(matches == 20).flatten()
matched_rmsds = rmsds[all_matched]

"""
Figures
"""
good_inds = torch.argwhere(opt_pred_rdfemd < 0.015).flatten()
density_funnel(opt_pred_rdfemd[good_inds],
               opt_cp[good_inds],
               rdf_dists[good_inds],
               ref_pred_rdfemd,
               ref_cp,
               yaxis_title='Predicted Distance',
               write_fig=True)
good_inds = torch.argwhere(opt_lj_energy < -350).flatten()
density_funnel(opt_lj_energy[good_inds],
               opt_cp[good_inds],
               rdf_dists[good_inds],
               ref_lj_energy,
               ref_cp,
               yaxis_title='LJ Energy (Arb Units)',
               write_fig=True)

compack_fig(matches, rmsds, write_fig=True)
```

The below file configures the optimization script `run_search.py`, with the target packing coefficient predicted via MXtalNet-D using the above-provided workflow.

```yaml
device: cuda
mol_path: /crystal_datasets/DAFMUV.pt
out_dir: /opt_outputs
score_model_checkpoint: checkpoints/crystal_score.pt
run_name: DAFMUV

mol_seed: 0
opt_seed: 0
sampling_mode: all
mols_to_sample: 1
num_samples: 1000

sgs_to_search: [33]
zp_to_search: [1]

batch_size: 1000
grow_batch_size: false

init_sample_method: random
init_target_cp: 0.699

```

```
opt:
  - optim_target: 'silu'
    enforce_niggli: false
    compression_factor: 1.0
    target_packing_coeff: 0.699
    init_lr: 0.001
    convergence_eps: 0.001
    optimizer_func: 'rprop'  # fastest and most reliable
    anneal_lr: false
    grad_norm_clip: 0.1
    show_tqdm: true
    max_num_steps: 500

  - optim_target: 'lj'
    enforce_niggli: false
    compression_factor: 0.0
    target_packing_coeff: 0.699
    init_lr: 1.0
    convergence_eps: 0.0001
    optimizer_func: 'sgd'
    anneal_lr: true
    grad_norm_clip: 0.01
    show_tqdm: true
    max_num_steps: 50
```
